# The Influence of Artificially Introduced N-Glycosylation Sites on the *In Vitro* Activity of *Xenopus laevis* Erythropoietin

**DOI:** 10.1371/journal.pone.0124676

**Published:** 2015-04-21

**Authors:** Kazumichi Nagasawa, Mizue Meguro, Kei Sato, Yuta Tanizaki, Nami Nogawa-Kosaka, Takashi Kato

**Affiliations:** 1 Integrative Bioscience and Biomedical Engineering, Graduate School of Advanced Science and Engineering, Waseda University, Center for Advanced Biomedical Science, TWIns building, 2–2 Wakamatsu-cho, Shinjuku-ku, Tokyo 162–8480, Japan; 2 Department of Biology, School of Education, Waseda University, Center for Advanced Biomedical Science, TWIns building, 2–2 Wakamatsu-cho, Shinjuku-ku, Tokyo 162–8480, Japan; King's College London, UNITED KINGDOM

## Abstract

Erythropoietin (EPO), the primary regulator of erythropoiesis, is a heavily glycosylated protein found in humans and several other mammals. Intriguingly, we have previously found that EPO in *Xenopus laevis* (*xl*EPO) has no *N*-glycosylation sites, and cross-reacts with the human EPO (huEPO) receptor despite low homology with huEPO. In this study, we introduced *N*-glycosylation sites into wild-type *xl*EPO at the positions homologous to those in huEPO, and tested whether the glycosylated mutein retained its biological activity. Seven *xl*EPO muteins, containing 1–3 additional *N*-linked carbohydrates at positions 24, 38, and/or 83, were expressed in COS-1 cells. The muteins exhibited lower secretion efficiency, higher hydrophilicity, and stronger acidic properties than the wild type. All muteins stimulated the proliferation of both cell lines, *xl*EPO receptor-expressing *xl*EPOR-FDC/P2 cells and huEPO receptor-expressing UT-7/EPO cells, in a dose-dependent manner. Thus, the muteins retained their *in vitro* biological activities. The maximum effect on *xl*EPOR-FDC/P2 proliferation was decreased by the addition of *N*-linked carbohydrates, but that on UT-7/EPO proliferation was not changed, indicating that the muteins act as partial agonists to the *xl*EPO receptor, and near-full agonists to the huEPO receptor. Hence, the EPO-EPOR binding site in *X*. *laevis* locates the distal region of artificially introduced three *N*-glycosylation sites, demonstrating that the vital conformation to exert biological activity is conserved between humans and *X*. *laevis*, despite the low similarity in primary structures of EPO and EPOR.

## Introduction

Erythropoietin (EPO) is a hematopoietic cytokine that regulates the rate of red blood cell production [1, 2]. It binds to EPO receptors (EPORs) on the surface of erythroid progenitors, and then causes a preformed EPOR homodimer to undergo a conformational change which activates signal transduction pathways. The signal transduction events promote the survival, proliferation, and differentiation of erythroid progenitors, causing an increase in the number of circulating mature red blood cells. In humans and mice, EPO is a heavily glycosylated protein produced by adult kidneys, and is targeted to the bone marrow via blood circulation. The carbohydrate chain plays a part in the prevention of EPO being cleared from blood circulation, and therefore is vital for *in vivo* biological activity [3–10].

Erythropoiesis is one of the common functions widely conserved among vertebrates. Thus far, EPO sequences have been elucidated in at least 13 mammalian species [11–20], one amphibian (African clawed frog, *Xenopus laevis*) [21], and 3 teleost fish (pufferfish, *Takifugu rubripes*; zebrafish, *Danio rerio*; and goldfish, *Carassius auratus L*.) [22–25]. Interspecific comparison of the functional region can provide an insight into the universal structure-function relationships among EPO proteins. The functional region responsible for the receptor binding and biological activity of human EPO (huEPO) is described by several studies [26–30]. However, in other species, especially non-mammals, the functional region of EPO remains unexplored.

Previously, we described the cloning of *X*. *laevis* EPO (*xl*EPO) and its *in vitro* activity by a colony formation assay [21, 31]. Intriguingly, we found that *xl*EPO stimulates the proliferation of the cells that express human EPOR (huEPOR), even though the amino acid sequence of *xl*EPO is only 38% identical to that of huEPO [21]. This led us to expect that the functional region of EPO, which is responsible for *in vitro* activity, is conserved between *X*. *laevis* and humans. In light of this assumption, there are several findings that helped us to generate a working hypothesis. First, *xl*EPO has no *N*-glycosylation site [21], whereas huEPO has 3 *N*-glycosylation sites (at Asn24, Asn38, and Asn83) that are distal to the receptor-binding site [32]. Remarkably, these 3 *N*-glycosylation sites [13] and their biological cross-reactivity [33–36] are conserved in a number of mammalian EPOs. In addition, it has been observed that in huEPO glycosylation analogs with additional *N*-linked carbohydrates, the positions of the carbohydrate chains have an impact on whether the analog retains its *in vitro* biological activity [32, 37]. In light of these facts, we hypothesized that the activity of *xl*EPO should be retained, even if we introduce *N*-glycosylation sites to regions homologous to the 3 *N*-glycosylation sites of huEPO.

In this study, we tested this hypothesis by examining the activity of *xl*EPO muteins containing artificial *N*-glycosylation sites at positions homologous to those in huEPO (i.e., Asn24, Asn38, and Asn83). Those *xl*EPO muteins retained *in vitro* activity, demonstrating that the fundamental conformations of EPO-EPOR binding are conserved among humans and *X*. *laevis*; despite the low similarity in the primary sequences.

### Notation of gene and protein symbols

The gene and protein symbols of *Xenopus* are suggested in Xenbase (Gene Nomenclature Guidelines; http://xenbase.org/gene/static/geneNomenclature.jsp). However, for reporting the comparative analysis of EPO in this study, we named the *X*. *laevis* erythropoietin protein “*xl*EPO” instead of xlepo.

## Materials and Methods

### Construction of *N-*glycosylated *xl*EPO muteins

**Fig 1:**
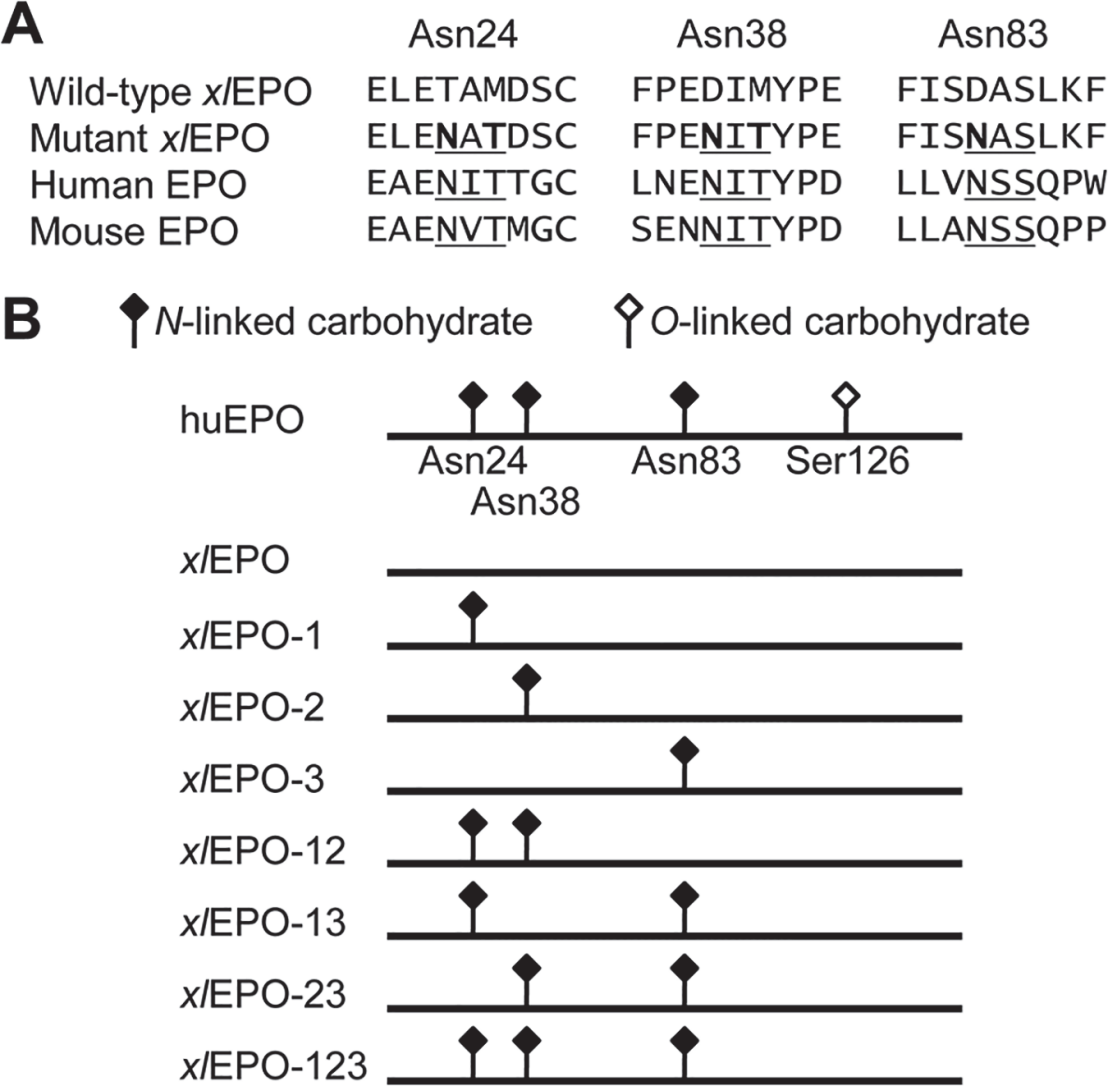
Design of *N*-glycosylated *xl*EPO muteins. (**A**) Schematic drawings of *Xenopus laevis* erythropoietin (*xl*EPO) muteins. *N*- and *O*-glycosylation sites are indicated by closed and opened rhombuses, respectively. The terms to the left of the bars represent the names of the muteins. “X” in *xl*EPO-XXX indicates *N*-glycosylation positions (1, Asn24; 2, Asn38; 3, Asn83). (**B**) Amino acid sequences around the introduced *N*-glycosylation sites in the *xl*EPO muteins. The parallel sequences of wild-type *xl*EPO, human EPO, and mouse EPO are also shown. Consensus sequences for *N*-glycosylation (Asn-Xxx-Ser/Thr) are underlined, with the amino acid substitutions in bold.

*N*-glycosylated *xl*EPO muteins were constructed by introducing *N*-glycosylation consensus sequences (Asn-Xxx-Ser/Thr, where Xxx is any amino acid except Pro) into a region homologous to that of huEPO. Each *N*-glycosylation site results from the mutation of Thr24 Met26 to Asn24 Thr26, Asp38 Met40 to Asn38 Thr40, and Asp83 to Asn83 (Fig 1A and Table 1). *N*-glycosylated *xl*EPO cDNAs were produced by *in vitro* multiple site-directed mutagenesis of the *xl*EPO cDNA, as described below.

**Table 1:** Primers used for multiple site-directed mutagenesis.

Primer	Oligonucleotide sequence (5′–3′)
Thr24Asn/Met26Thr	AGAGAATTGGAAA**AC**GCCA**C**GGACTCCTGCAAC^*a*^
Asp38Asn/Met40Thr	CAATTTCCTGAG**A**ATATCA**C**GGTCCCTGAAAC^*a*^
Asp83Asn	ATTTCATCTCC**A**ATGCCAGCCTC^*a*^
tailed 5′-anchor	*ACTTGCGGATCCATGGC*GCTAGCCACCATGGGTGT^*b*^
tailed 3′-anchor	GCTAGTCTACCAACGTGA *CGGTCGTTAACAGCTCT* ^*b*^
PCR 5′-anchor Fw	ACTTGCGGATCCATGGC
PCR 3′-anchor Re	AGAGCTGTTAACGACCG

^*a*^The codons for mutated amino acids are underlined, with the point mutations in bold.

^*b*^The start and stop codons are underlined and the unique nucleotide tail for subsequent mutant strand-specific PCR is in italics.

Fw, forward; Re, reverse.

### Multiple site-directed mutagenesis and preparation of expression plasmids

Mutagenesis was performed as described by Seyfang and Jin [38], with some modifications. Double-stranded plasmid DNA containing the *xl*EPO gene [21] was used as a template. Mutagenic primers and tailed 5′- and 3′-anchor primers (Table 1) were phosphorylated before the annealing reaction. Two terminal-tailed primers with a unique 17-nucleotide tail (Table 1) were simultaneously annealed to the template DNA along with the set of mutagenic primers. Mutant strands were synthesized by primer extension and ligation in a single incubation step using T4 DNA polymerase and ligase. Full-length mutant strands were subsequently amplified by high-fidelity PCR with *Pfu* polymerase, which uses the unique mutant strand-specific tails introduced by the 2 terminal anchor primers. The specificity and band size of the amplified mutagenesis products were confirmed by 1.5% agarose gel electrophoresis. Following gel extraction and purification (QIAEX II Gel Extraction Kit; QIAGEN K.K., Tokyo, Japan) of the 584-bp band, the extracted DNA was cloned into a pGEM-T Easy vector (Promega K.K., Tokyo, Japan) and sequenced using an ABI3100 Genetic Analyzer (Applied Biosystems) and dye-terminator chemistry (BigDye Terminator; Applied Biosystems). Plasmids containing the desired mutations were digested with *Nhe*I and *Eco*RI. The DNA fragments were then cloned into the multiple cloning site (MCS) of the pIRES2-EGFP expression vector (BD Biosciences Clontech) containing the human cytomegalovirus immediate early promoter and an SV40 early polyadenylation signal sequence. pIRES2-EGFP contains the internal ribosome entry site (IRES) of the encephalomyocarditis virus between the MCS and the EGFP coding region; this enables both the *xl*EPO gene and the EGFP gene to be translated from a single bicistronic mRNA.

All constructs were transformed into *E*. *coli* DH5*α*. Several colonies were subsequently grown in Luria-Bertani broth containing 50 μg/mL of kanamycin. The plasmids were isolated using the alkaline-SDS lysis method.

### Expression of *xl*EPO muteins in COS-1 cells

Monkey kidney fibroblast COS-1 cells (Cell Bank, RIKEN BioResource Center, Ibaraki, Japan) were transfected with vectors that incorporated the full coding sequence of the *xl*EPO genes. COS-1 cells were cultured in Dulbecco’s modified Eagle’s medium (DMEM) supplemented with 10% (v/v) FBS, 100 U/mL penicillin, and 100 μg/mL streptomycin in a humidified atmosphere of 5% CO_2_ at 37°C. Cells (8 × 10^4^ cells/well) were seeded in 24-well cell culture plates (Corning Incorporated, NY, USA). When the cultures reached 90% confluence, cells were transfected with a mixture of 0.8 μg plasmid cDNA and 1.5 μL Lipofectamine LTX (Life Technologies Corporation) per well in 100 μL Opti-MEM (Life Technologies Corporation) for 4 h at 37°C. After transfection, cells were incubated in antibiotic-free DMEM containing 10% FBS for 24 h. Cells were then incubated in serum-free, antibiotic-containing DMEM. After an additional 48 h, EGFP fluorescence was measured (excitation 485 nm, emission 528 nm) and conditioned media were collected. The collected media were arbitrarily concentrated using centrifugal filter devices (Amicon Ultra-4, 5000 NMWL; Millipore Corporation, Billerica, MA, USA) and stored at—80°C. Expression of the *xl*EPO muteins was confirmed by SDS-PAGE followed by Western blotting.

### Gel electrophoresis and Western blot analyses

Samples were reduced by boiling for 5–10 min in sample buffer (50 mM Tris-HCl (pH 6.8), 20 mM dithiothreitol, 1% w/v SDS, 10% v/v glycerol, 2 mM EDTA, and 0.005% bromophenol blue) and subjected to SDS-PAGE with a separating gel containing 14% acrylamide. Protein markers (Biotinylated SDS-PAGE Standard Low Range; Bio-Rad Laboratories Inc.) were used for electrophoretic estimation of molecular weights. After electrophoresis, the proteins were transferred to a polyvinylidene difluoride membrane (Millipore). Protein transfer was carried out for 30 min with a constant current of 150 mA in a semi-dry electroblotter (Continental Lab Products Inc., San Diego, CA, USA) using anolyte (0.3 M Tris (pH 10.4) and 20% v/v methanol), transfer membrane (25 mM Tris (pH 10.4) and 20% v/v methanol), and catholyte solutions (25 mM Tris (pH 10.4) and 40 mM 6-amino-*n*-caproic acid, 20% v/v methanol). The blots were washed once with TBS (20 mM Tris-HCl (pH 7.5) and 500 mM NaCl) and then twice with TBS containing 0.1% (v/v) Tween 20 (TTBS). After blocking with 0.4% Block Ace (Snow Brand Milk Products Co., Ltd., Hokkaido, Japan) in TTBS for 45 min, each blot was incubated for 1 h with 2 μg/mL anti-peptide antibody against the *xl*EPO Thr44 to Ser57 sequence (anti-*xl*EPO sequence antibody) [31] or anti-GFP antibody (Medical & Biological Laboratories Co., Ltd., Nagoya, Japan) and then washed twice with TTBS. The blots were subsequently incubated for 1 h with biotinylated goat anti-rabbit IgG (1:6000; Bio-Rad), washed twice with TTBS, and treated with alkaline phosphatase-conjugated StrepTactin (1:6000; Bio-Rad) for 1 h. After 2 washes with TTBS followed by 2 more washes with TBS, proteins were detected by chemiluminescence (CDP-Star reagent; GE Healthcare Ltd., Tokyo, Japan). The bands were detected using a Luminescent Image analyzer LAS-3000 (Fuji Photo Film Co., Ltd., Tokyo, Japan). All washes were carried out for 5 min. All antibodies and alkaline phosphatase-conjugated StrepTactin were prepared in TTBS containing 0.4% Block Ace.

### Physicochemical analysis of *xl*EPO muteins

Wild-type *xl*EPO and *N*-glycosylated muteins in mixed conditioned media were separated by reverse-phase high-pressure liquid chromatography (RP-HPLC) and cation exchange chromatography (CIEC). In RP-HPLC, the sample was applied at a flow rate of 0.4 mL/min to a YMC-Pack Protein-RP column (4.5 × 150 mm; YMC Co., Ltd., Tokyo, Japan) and separated with a linear gradient of 20%–60% acetonitrile in 0.1% trifluoroacetic acid over 30 min. A total of 10 fractions was collected once every 3 min (1.2 mL). In CIEC, the samples were adjusted to pH 2 with acetic acid, applied to 200 μL of SP Sepharose FF column (GE Healthcare), and eluted with 50 mM citrate/100 mM phosphate buffer (pH 2, 3, 4… 9). Each fraction was subjected to Western blot analysis.

### De-*N*-glycosylation of *xl*EPO muteins

*N*-linked carbohydrates were removed by incubation with or without 0.8 U peptide *N*-glycosidase F (PNGase F; Roche Diagnostics K.K., Tokyo, Japan) in 20 mM sodium phosphate buffer (pH 8.6) containing 10 mM EDTA at 37°C for 24 h. Carbohydrate removal was assessed by SDS-PAGE and Western blot analysis.

### Cell proliferation assay

Cell proliferation assays were performed as previously described [21]. Two cell lines were used: the interleukin-3-dependent murine cell line FDC/P2, which expresses the exogenous *xl*EPO receptor, *xl*EPOR-FDC/P2 [21], and the EPO-dependent human cell line, UT-7/EPO [39]. In the MTS assay, absorbance at 450 nm was measured using a microplate reader (POWERSCAN HT; DS Pharma Biomedical Co., Ltd., Osaka, Japan). Dose-response curves were fitted to a 4-parameter logistic model: *y* = (*a—d*) / {1 + (*x* / *c*) *b*} + *d*, where, *a* is *E*_max_ and *c* is *EC*_50_ [40]. Then, the *E*_max_ (maximum effect) of the *xl*EPO mutein was determined without normalization by protein amount. The relative *EC*_50_ (half-maximal effective concentration) of the *xl*EPO mutein was determined via normalization of v/v % of COS-1 supernatant *EC*_50_ with its relative amount in COS-1 supernatant.

### Statistical analysis

All experiments were performed at least 3 times with triplicate samples, and similar results were obtained each time. Error bars represent the standard deviations (SD). Either Student’s *t*-test or the Tukey–Kramer test was used, when appropriate.

## Results

### Design and expression of *N*-glycosylated *xl*EPO muteins

**Fig 2:**
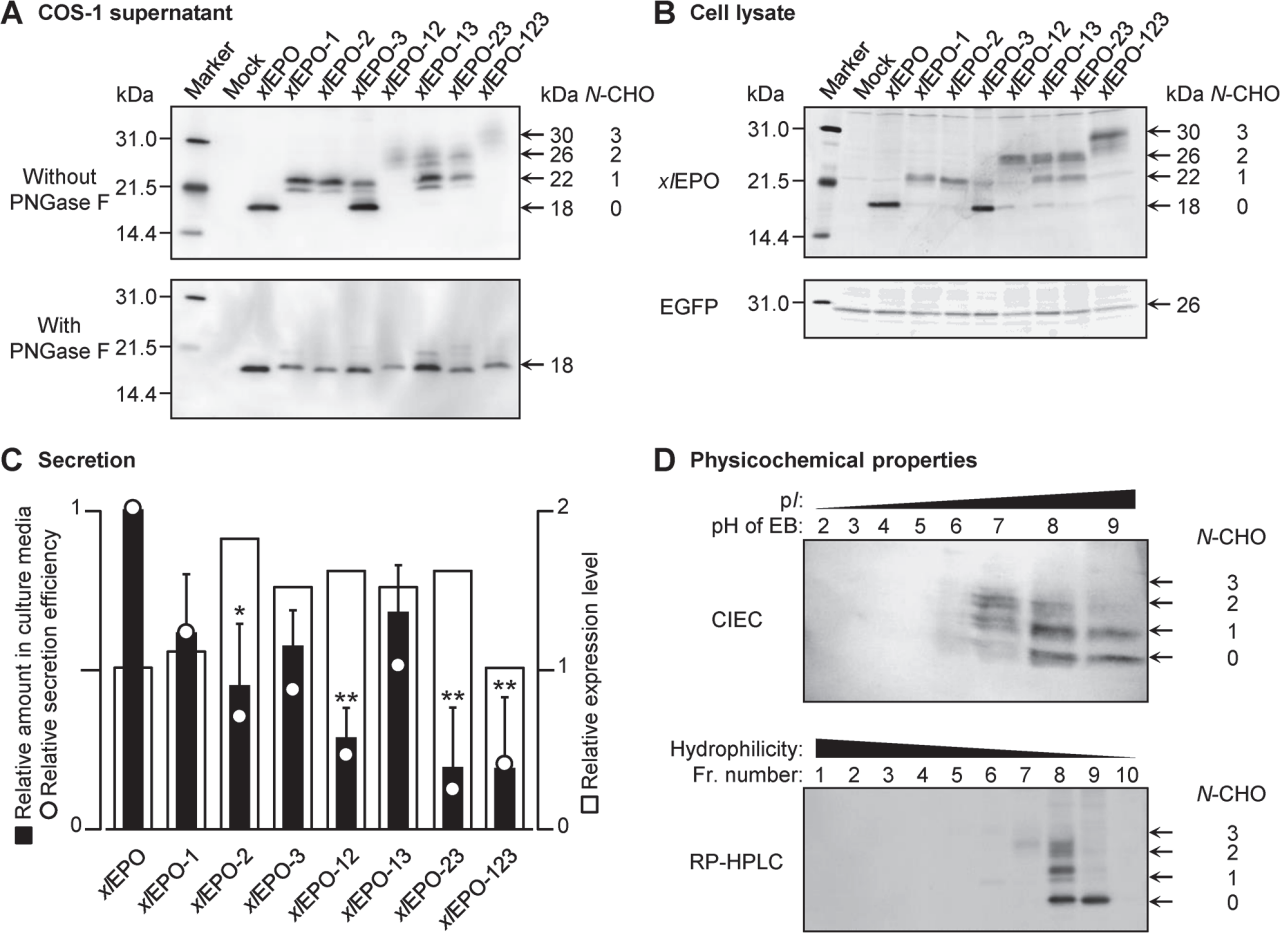
Expression, secretion, and physicochemical properties of *N*-glycosylated *xl*EPO muteins. (**A**) Western blot analysis of *xl*EPO muteins secreted from COS-1 cells. Conditioned media were concentrated 10-fold (*top panel*), and subjected to PNGase F treatment (*bottom panel*). Wild-type *xl*EPO and *N*-glycosylated muteins were separated by SDS-PAGE (reducing conditions) and visualized by Western blot analysis. Equal volumes of samples were loaded into each lane. (**B**) Western blot analysis of *xl*EPO muteins remaining in COS-1 cells. Whole-cell lysates were loaded into each lane. *xl*EPO (*top panel*) and EGFP (*bottom panel*) were visualized by Western blot analysis. Co-expressed nonsecretory EGFP was used as a loading control. (A, B) Standard proteins are shown at the far left, and the letters above each lane represent the names of the muteins shown in Fig 1. (**C**) Effects of *N*-glycosylation on the secretion of *xl*EPO muteins. The relative amounts of *xl*EPO muteins in the culture media of COS-1 cells were measured by densitometry analysis of Western blots (*filled bars*). The relative expression levels were calculated based on the fluorescence intensity of co-expressed EGFP (*open bars*). The relative efficiency of secretion was obtained by the division of relative amount by relative expression level (*open circles*). Values were normalized to that of the wild type, which was set at 1. *p < 0.05 and **p < 0.01 compared to the wild type. (**D**) Physicochemical properties of *xl*EPO muteins. Cation-exchange chromatography (CIEC, *top panel*) and the reverse-phase high-pressure liquid chromatography (RP-HPLC, *bottom panel*) of wild-type *xl*EPO and *N*-glycosylated muteins were performed as described in the *Materials and methods* section. The collected fractions were subjected to Western blot analysis. ‘*N*-CHO’ indicates the number of *N*-linked carbohydrates. Abbreviations are: EB, elution buffer; Fr., fraction.

We introduced *N*-glycosylation consensus sequences into the *xl*EPO at positions homologous to those of huEPO (i.e., Asn24, Asn38, and Asn83) using *in vitro* mutagenesis (Fig 1A). The mutations were introduced both individually and in combinations. Thus, we constructed 7 different *N*-glycosylated *xl*EPO muteins with consensus sequences introduced at 3 different positions (Fig 1B). The wild-type *xl*EPO and *N*-glycosylated muteins were expressed in COS-1 cells using the lipofection method. All muteins were secreted from the COS-1 cells. The extent of glycosylation was evaluated by Western blot analysis using the anti-*xl*EPO sequence antibody (Fig 2A, *top panel*). The molecular weight of wild-type *xl*EPO was 18 kDa, which is equivalent to that of the peptide backbone. Western blots showed progressive increases in the molecular weights of *xl*EPO muteins from 18 to 22, 26, and 30 kDa, depending on the number of *N*-linked carbohydrates added. Treatment with PNGase F, which removes *N*-linked carbohydrates from glycoproteins, shifted the molecular weights of all muteins to 18 kDa, or the molecular weight of the wild type (Fig 2A, *bottom panel*). Thus, the increase in the size of the muteins in comparison to the wild-type *xl*EPO was attributed to *N*-linked carbohydrate content. Non-glycosylated Asn83 was observed in *xl*EPO-3, *xl*EPO-13, *xl*EPO-23, and *xl*EPO-123, which indicates that carbohydrate additions were efficient at Asn24 and Asn38, but inefficient at Asn83. The glycosylated *xl*EPO molecules showed 2 bands: a main band and a smaller one. These 2 bands were not observed after PNGase F treatment. This result shows that these 2 bands result from heterogeneity in the *N*-linked carbohydrate structure. The unsecreted protein was also examined by Western blot analysis (Fig 2B). Whole-cell lysates were loaded into each lane, and EGFP was used as a loading control to normalize the signals (Fig 2B, *bottom panel*). The wild-type *xl*EPO and *N*-glycosylated muteins left in the cell were detected (Fig 2B, *top panel*). To estimate the amount of *xl*EPO muteins in the COS-1 supernatant, we performed a densitometry analysis of Western blots. In Western blot analysis, the recognition of hyperglycosylated huEPO analogs by an anti-huEPO sequence antibody was reported to be weak compared with huEPO recognition, and was improved by the partial elimination of carbohydrate chains [41]. Therefore, we used Western blots of deglycosylated samples (Fig 2A, *bottom panel*) for densitometry analysis, because the added *N*-linked carbohydrates may prevent the anti-*xl*EPO sequence antibody from binding to the *xl*EPO muteins. The band intensity of wild-type *xl*EPO was defined as 1. The analysis revealed that the relative amount of *xl*EPO muteins in the COS-1 supernatant decreased depending on the number of *N*-linked carbohydrates added (Fig 2C). The detected amounts of the muteins, *xl*EPO-1, *xl*EPO-2, and *xl*EPO-3, containing 1 *N*-glycosylation site were approximately half that of the wild type. Moreover, the amounts of the muteins, *xl*EPO-12, *xl*EPO-23, and *xl*EPO-123, containing 2 or 3 *N*-glycosylation sites were only about one-quarter that of the wild type. Because the IRES-containing expression vector enables *xl*EPO and EGFP to be co-expressed from a single bicistronic mRNA, EGFP fluorescence intensity reflects *xl*EPO expression at the mRNA level. Therefore, the relative secretion efficiency of *xl*EPO muteins was obtained by dividing the relative amount (i.e. relative secretion level) by the relative expression level (Fig 2C). As a result, the secretion efficiencies of *xl*EPO muteins were revealed to decrease as the number of *N*-linked carbohydrates added increased, suggesting that glycosylation may have an adverse effect on *xl*EPO secretion.

### Physicochemical properties of *xl*EPO muteins

The physicochemical properties of proteins, such as the electrical charge and hydrophilicity, affect their ligand–receptor binding affinity and/or protein stability in blood [42, 43]. To predict the isoelectric point (p*I*) of the *xl*EPO muteins, CIEC was performed (Fig 2D, *top panel*). Wild-type *xl*EPO was eluted at pH 8–9, and the *xl*EPO muteins containing 1 or 2 *N*-linked carbohydrates were eluted mainly at pH 7 or 8, respectively. Next, RP-HLPC was performed, in order to examine the hydrophilicity of the *xl*EPO muteins (Fig 2D, *bottom panel*). Wild-type *xl*EPO was eluted in fraction (Fr.) 9 at 52%–58% acetonitrile. Meanwhile, *xl*EPO muteins with 1 or 2 *N*-linked carbohydrates were eluted in earlier fractions (Fr. 8, 46%–52% acetonitrile or Fr. 7, 40%–46% acetonitrile, respectively). These results indicate that the *xl*EPO muteins are more acidic and hydrophilic than the wild type, owing to the negative charge of *N*-linked carbohydrates.

### *In vitro* activity of *xl*EPO muteins

**Fig 3:**
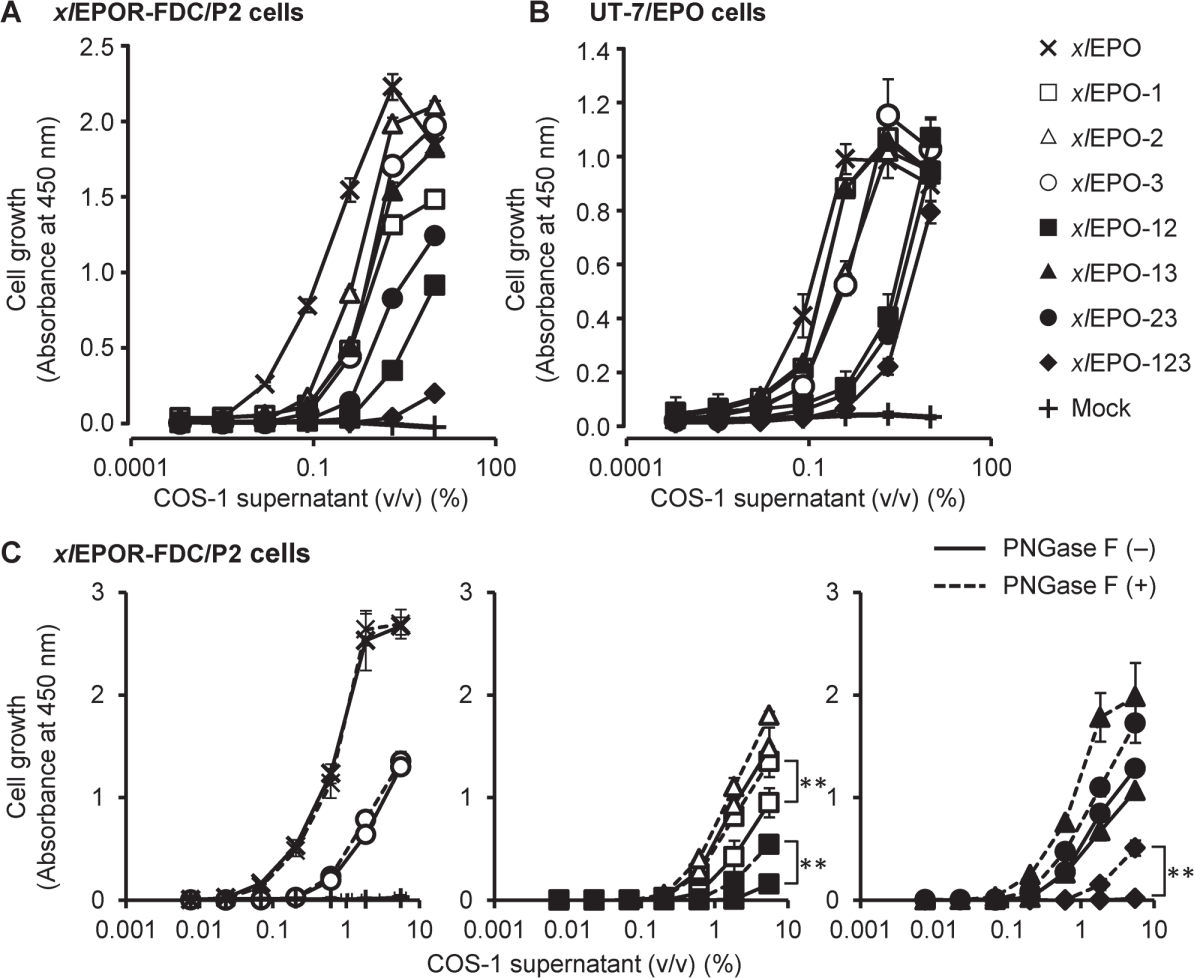
Effects of wild-type *xl*EPO and the glycosylated muteins on the proliferation of *X*. *laevis* and human EPOR-expressing cells. (**A, B**) The proliferative responses of *xl*EPOR-FDC/P2 cells (A) and UT-7/EPO cells (B) to the wild-type *xl*EPO and the *N*-glycosylated muteins were assessed using the MTS assay. Incubations were performed in triplicate, and the results are presented as the mean (± SD) from 1 of 4 independent experiments. (**C**) The proliferative responses of *xl*EPOR-FDC/P2 cells to wild-type *xl*EPO and the *N*-glycosylated muteins treated with (*dashed line*) and without (*solid line*) PNGase F were also assessed. The PNGase F-treated samples are the same fractions displayed in Fig 2A, *bottom panel*. Incubations were performed in triplicate, and the results are presented as the mean (± SD) from 1 of 3 independent experiments. **p < 0.01 for the comparison. Symbols are: *multiple marks*, wild-type *xl*EPO; *open squares*, *xl*EPO-1; *open triangles*, *xl*EPO-2; *open circles*, *xl*EPO-3; *closed squares*, *xl*EPO-12; *closed triangles*, *xl*EPO-13; *closed circles*, *xl*EPO-23; *closed rhombuses*, *xl*EPO-123; *plus sign*, Mock (control culture supernatant without *xl*EPO).

**Fig 4:**
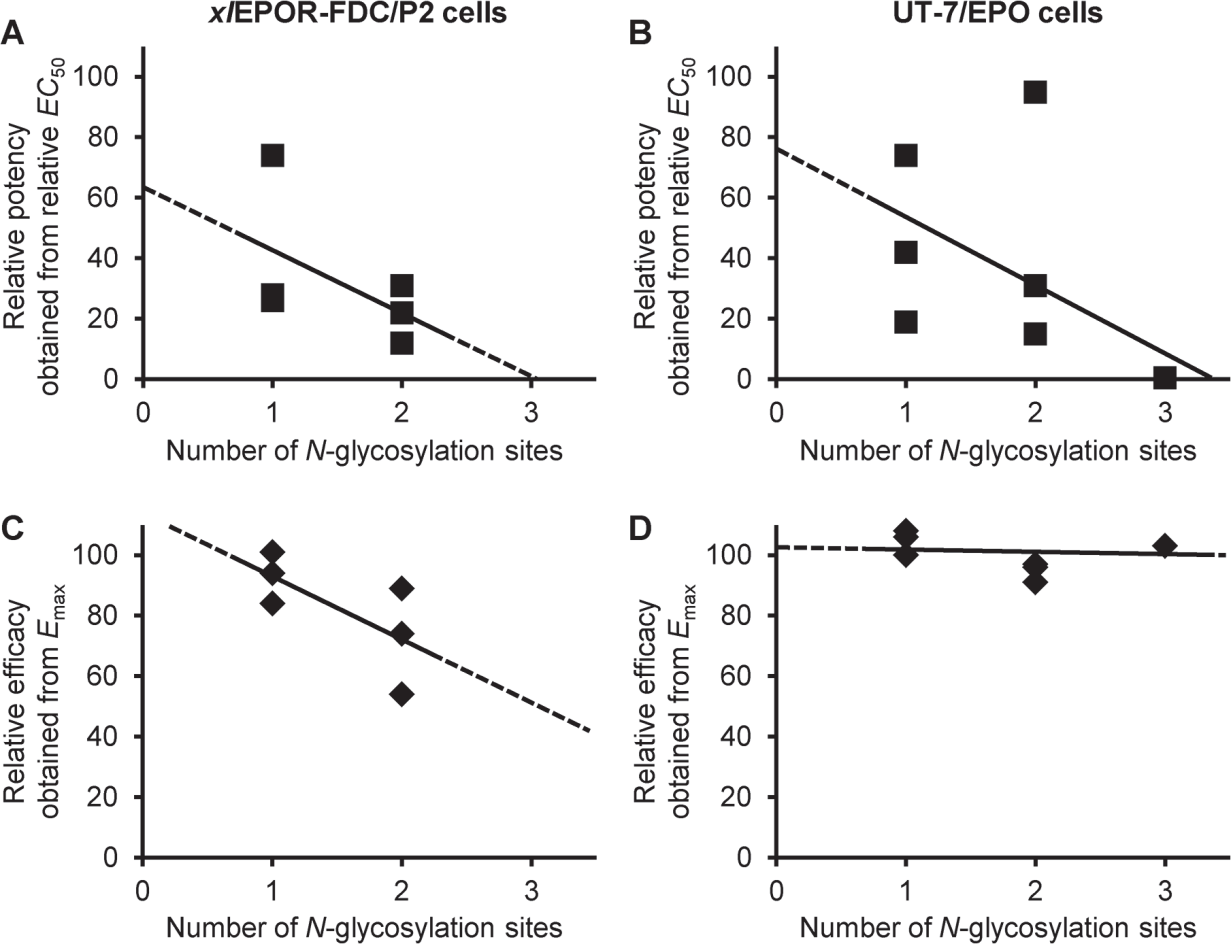
Relationship between carbohydrate content and biological activity. The COS-1 supernatants containing the wild-type *xl*EPO and the *N*-glycosylated muteins were assayed by proliferation of *xl*EPOR-FDC/P2 cells (A, C) and UT-7/EPO cells (B, D). *EC*_50_-derived relative potency (A, B) and *E*_max_-derived relative efficacy (C, D) are shown as a percentage relative to the wild-type *xl*EPO (Table 2) and are plotted against the number of added *N*-glycosylation sites. Each point represents different *xl*EPO muteins.

The effects of the added *N*-linked carbohydrates on the *in vitro* activity of *xl*EPO muteins were tested in cell proliferation assays using exogenous *xl*EPOR-expressing *xl*EPOR-FDC/P2 cells (Fig 3A), and huEPOR-expressing UT-7/EPO cells (Fig 3B). As expected, all *xl*EPO muteins stimulated the proliferation of both cell lines in a dose-dependent manner. Usually, dose-response curves as descriptors of ligand activity are characterized by differences in potency (described as *EC*_50_) and efficacy (described as *E*_max_). Then, the relative potency and the relative efficacy of the *xl*EPO muteins on the proliferation of *xl*EPOR-FDC/P2 and UT-7/EPO cells were evaluated (Table 2 and Fig 4). As the number of *N*-linked carbohydrates increased, the relative potency of *xl*EPO muteins, which was obtained from the relative *EC*_50_, tended to decrease in both cell lines (Fig 4A and 4B). On the other hand, the effect of *N*-linked carbohydrates on the relative efficacy of *xl*EPO muteins, which was obtained from *E*_max_, differed between the cell lines (Fig 4C and 4D). The efficacy on the proliferation of *xl*EPOR-FDC/P2 cells tended to decrease, as the number of *N*-linked carbohydrates increased (Fig 4C). More specifically, the efficacy of *xl*EPO-12 and *xl*EPO-23 was significantly lower than that of wild-type *xl*EPO, and *xl*EPO-1 and *xl*EPO-13 also showed a slightly lower efficacy compared to the wild type (Table 2). In contrast, the efficacy of the *xl*EPO muteins on the proliferation of UT-7/EPO cells was not significantly affected by the addition of *N*-linked carbohydrates (Fig 4D and Table 2). To confirm that the decrease in the activity of *xl*EPO muteins (*EC*_50_ and *E*_max_) is attributed to the addition of *N*-linked carbohydrates, PNGase F-treated COS-1 supernatant was assayed by the proliferation of *xl*EPOR-FDC/P2 cells (Fig 3C). Removal of *N*-linked carbohydrates with PNGase F increased the *in vitro* activity of the *xl*EPO muteins (Fig 3C, *middle and right panels*), except *xl*EPO-3 (Fig 3C, *left panel*), when COS-1 supernatants were added at a final concentration of 6%. This indicates that the added *N*-linked carbohydrates decreased the *in vitro* activity of *xl*EPO.

**Table 2:** Relative *in vitr*o activities of *xl*EPO muteins.

	Relative *EC*_50_ ^*a*^ (relative potency, %)^*b*^		Relative efficacy_,_ %^*c*^	
Protein	*xl*EPOR-FDC/P2	UT-7/EPO	*xl*EPOR-FDC/P2	UT-7/EPO
*xl*EPO-1	3.84 ± 0.54 (26)	1.34 ± 0.27 (74)	84 ± 15	106 ± 9
*xl*EPO-2	1.35 ± 0.79 (74)	5.17 ± 4.07 (19)	101 ± 5	108 ± 27
*xl*EPO-3	3.54 ± 1.50 (28)	2.37 ± 0.48 (42)	94 ± 6	100 ± 14
*xl*EPO-12	8.65 ± 1.58** (12)	3.24 ± 2.88 (31)	54 ± 16**	96 ± 8
*xl*EPO-13	4.47 ± 2.97 (22)	1.05 ± 0.43 (95)	89 ± 8	97 ± 20
*xl*EPO-23	3.25 ± 1.41 (31)	6.64 (15)	74 ± 14*	91
*xl*EPO-123	N.D.	187 (0.54)	N.D.	103

^*a*^The relative half-maximal effective concentrations (*EC*_50_) of the *xl*EPO muteins. *EC*_50_ of wild-type *xl*EPO was defined as 1. Values are presented as mean ± SD of 2–4 independent experiments.

^*b*^The relative potencies are expressed as the value of the wild type equal to 100%.

^*c*^The relative efficacy of the *xl*EPO muteins. Values were obtained from maximum effects (*E*_max_) as the value of wild-type *xl*EPO equal to 100%. Values are presented as mean ± SD of 2–4 independent experiments.

*p < 0.05 and

**p < 0.01 for the muteins compared with the wild type.

N.D., not determined.

## Discussion

In this study, we produced 7 different *N*-glycosylated *xl*EPO muteins and compared their biochemical properties of (i) secretion, (ii) physicochemical properties, and (iii) *in vitro* activity to those of the wild type.

The relative secretion efficiencies of the *xl*EPO muteins decreased depending on the number of *N*-linked carbohydrates added (Fig 2C). When the carbohydrate is added to the partially folded protein during translation [44], it has been hypothesized that the rates of synthesis or folding have a competitive relationship with the addition of the carbohydrate [45]. In light of this, the addition of carbohydrates was thought to reduce the secretion of *xl*EPO muteins at translation and/or during the secretion process.

Wild-type *xl*EPO was absorbed to the cation exchanger at pH 7 (Fig 2D). The pH of *X*. *laevis* plasma is approximately 7.4, the same as that of human plasma [46]. This indicates that wild-type *xl*EPO bears a positive electric charge in blood, whereas huEPO (p*I*, 4.4–5.1) bears a negative charge [47]. In case of huEPO, sialic acid of carbohydrates, which is negatively charged at physiological pH, is thought to decrease EPOR binding via charge repulsion. Accordingly, wild-type *xl*EPO is thought to easily access EPORs on cells compared to huEPO.

All *N*-glycosylated *xl*EPO muteins retained *in vitro* activity against the cell proliferation of both *xl*EPOR-FDC/P2 and UT-7/EPO (Fig 3A and 3B). The *EC*_50_ and *E*_max_ of a ligand represent the ability of the ligand to bind to its receptor (i.e., potency which reflects receptor-binding affinity) and its ability to cause an effect after binding to the receptor (i.e., efficacy which is intrinsic acticity), respectively. In the present study, we showed the relative *EC*_50_ because we were able to roughly quantify the relative amounts of wild-type *xl*EPO and *N*-glycosylated muteins in conditioned media. As a result, we found that the addition of *N*-linked carbohydrates decreased the *EC*_50_-derived relative potency of *xl*EPO muteins (Fig 4A and 4B). It has been reported that *N*-linked carbohydrates reduce the affinity of huEPO for its receptor, as well as the *in vitr*o activity of huEPO [5]. This can be explained as follows: the negatively charged sialic acid and carbohydrate individually decrease the association rate constant of huEPO with huEPOR *via* their negative effect on intrinsic electrostatic enhancement [42]. It is also known that darbepoetin alfa, a hyperglycosylated huEPO analog with 2 extra *N*-linked carbohydrates, shows lower receptor-binding activity than huEPO [48]. Here, we confirmed that the *N*-glycosylated *xl*EPO muteins are more negatively charged than the wild type (Fig 2D). Taken together, it is thought that one of the reasons for reduced potencies of muteins is their lower receptor-binding affinities than that of wild type due to negative charge of carbohydrates.

Type of ligand effect on a receptor can be classified according to its behavior. Full agonists elicit the same level of full effect (i.e. *E*_max_, efficacy) as the natural ligand of the receptor, while partial agonists also cause an effect, but they cannot reach the same level of the natural ligand. The efficacy of the *xl*EPO muteins on *xl*EPOR-FDC/P2 cell proliferation was less than that of wild-type *xl*EPO (Fig 4C). In particularly, the efficacy of the *xl*EPO muteins containing *N*-linked carbohydrates on Asn24 was remarkably lower than that of wild-type *xl*EPO (Table 2). These data indicate that the muteins act as partial agonists against *xl*EPOR, and also suggest that the carbohydrate structure located at Asn24 interferes with *xl*EPOR binding. In contrast, the efficacy of *xl*EPO on the proliferation of UT-7/EPO cells was not changed by the addition of *N*-linked carbohydrates (Fig 4D). The *E*_max_ of wild-type *xl*EPO and the *N*-glycosylated muteins was approximately the same as that of huEPO. These data indicate that the muteins act as near-full agonists against huEPOR, and also suggest that the *N*-linked carbohydrates at the Asn24, Asn38, and Asn83 sites of the *xl*EPO muteins do not interfere with huEPOR binding. Analysis of the effect of point mutations on bioactivity [26–29] and the crystal structure of huEPO, complexed with the extracellular ligand-binding domains of huEPOR [30], have identified amino acid residues important for receptor binding and biological activity. Twelve of the 18 amino acid residues that reduce the *in vitro* bioactivity of huEPO following point mutations, and interact with huEPOR residues, are conserved in *xl*EPO (6 of 10 in site 1, 6 of 8 in site 2), despite an only 38% overall sequence identity between *xl*EPO and huEPO (Table 3) [21]. These highly conserved amino acid residues are thought to allow *xl*EPO to cross-react with huEPOR and retain its proper orientation, avoiding interference from the added *N*-linked carbohydrates. With regard to *xl*EPOR, 7 of the 13 amino acid residues of huEPOR that interact with huEPO residues are conserved in *xl*EPOR (6 of 8 in site 1, 2 of 8 in site 2), which has an only 26% sequence identity with huEPOR in the extracellular domain (Table 3) [49]. Further investigation is needed to understand why the added carbohydrates on *xl*EPO interfere with the *xl*EPOR binding but not with the huEPOR binding.

**Table 3:** Amino acid residues of huEPO and huEPOR that contact each other in site 1 and site 2 intermolecular contact areas, and the corresponding residues in *xl*EPO.

Site 1				Site 2			
*xl*EPO residue^*a*^	huEPO residue^*b*^ ^,^ ^*d*^	Neighboring huEPOR residues^*c*^ ^,^ ^*d*^	Conserved *xl*EPOR residues^*e*^	*xl*EPO residue^*a*^	huEPO residue^*b*^ ^,^ ^*d*^	Neighboring huEPOR residues^*c*^ ^,^ ^*d*^	Conserved *xl*EPOR residues^*e*^
K9	*S9*	*H153*		P5	*L5*	***F93***, **S204**	F85, S195
T10	*R10*	*H153*, *E176*		**D8**	*D8*	*H153*	
D13	E13	P203		T10	R10	M150, S152, H153	
I16	L16	P203, **S204**	S195	**V11**	***V11***	***F93***, M150, H153, **S204**	F85, S195
K17	L17	P203		M14	***R14***	***L33***, E34, P149, M150	L31
R20	**K20**	E202, P203		F15	***Y15***	S92, ***F93***	F85
**T44**	***T44***	S92, ***F93***, V94	F85	T78	Q78	A88	
**K45**	***K45***	*E62*, S91, S92, **F93**, V94	F85	E91	D96	T87, A88	
L46	***V46***	T90, S91, S92, ***F93***	F85	**K92**	***K97***	*E34*	
**N47**	N47	T87, A88, **D89**, T90, S91	D81	H94	V99	A88	
V48	***F48***	L33, E34, S92, ***F93***, F205	F85	**S95**	***S100***	T87, A88, T90, *S91*	
G49	Y49	E34, T87		**R98**	***R103***	L59, *E62*, *A88*, **D89**, *S91*, V94	D81
N52	K52	E34		**S99**	***S104***	S91, *S92*, ***F93***	F85
K121	R131	**E60**, **D61**, E62	E52, D53	H102	*T107*	***F93***, V94	F85
L123	I133	**D61**	D53	**L103**	***L108***	***F93***	F85
**K130**	*K140*	***E60***, ***D61***	E52, D53	K105	**R110**	**E60, D61**	E52, D53
S133	***R143***	***E60***, P95	E52				
**N137**	***N147***	***F93***, V94, P95, H114	F85				
**R140**	***R150***	***F93***, H114, **N116**, ***E117***, P203, **S204**	F85, N107, E108, S195				
**G141**	***G151***	***F93***	F85				
R144	K154	H153, **S204**	S195				
**L145**	***L155***	***F93***	F85				

^*a*^ *xl*EPO residues corresponding to right-hand “huEPO residues” [21]. Amino acid residues that are identical to huEPO residues are in bold.

^*b*^Buried residues (>5.0 Å^2^) of huEPO in site 1 and site 2 intermolecular contact areas [30]. Available mutagenesis information [26–29] are highlighted according to the maximal degree of loss of *in vitro* bioactivity: bold underlined, >50-fold; bold, >5-fold; underlined, 2–5-fold; not highlighted, no effect.

^*c*^HuEPOR residues within 4.5-Å distance from huEPO in the crystal structure of human EPO-(EPOR)_2_ complex (Protein Data Bank accession number, 1EER) [30]. Amino acid residues that are conserved in *X*. *laevis* are in bold [49].

^*d*^Amino acid residues of huEPO and huEPOR that form interactions with each other, such as hydrogen bond and hydrophobic binding, are in italics [30].

^*e*^Amino acid residues of *xl*EPOR that conserved in huEPOR[49].

huEPO, human erythropoietin; huEPOR, human EPO receptor; *xl*EPO, *X*. *laevis* EPO.

The most important finding presented here is that the carbohydrate structures, which are added to those *xl*EPO sites homologous to the *N*-glycosylation sites of huEPO, do not completely eliminate the *in vitro* biological activity of *xl*EPO, namely its binding to EPOR. This suggests that the functional region of *xl*EPO, which is responsible for its *in vitro* activity, must be located in a region other than the introduced *N*-glycosylation sites, as the 3 *N*-glycosylation sites of huEPO are distal to the receptor-binding site. We therefore conclude that a fundamental conformation of EPO-EPOR binding could be conserved between *X*. *laevis* and humans. Further point mutation study at the *xl*EPO-*xl*EPOR/huEPOR interface will give definite conclusions about their active sites. Furthemore, the key to understanding the observations presented here, as well as this conclusion, is the elucidation of the crystal structure of *xl*EPO in a complex with the extracellular domains of *xl*EPOR. Cross-species comparison of the tertiary structures of *X*. *laevis* and human EPOs (and other species such as fish) will facilitate the identification of the common denominators present in vertebrate EPOs.

## References

[1] Jelkmann W. (2013) Physiology and pharmacology of erythropoietin. Transfus Med Hemother 40: 302–309. 10.1159/000356193 24273483 PMC3822280

[2] Bunn HF. (2013) Erythropoietin. Cold Spring Harb Perspect Med 3: a011619 10.1101/cshperspect.a011619 23457296 PMC3579209

[3] Fukuda MN, Sasaki H, Lopez L, Fukuda M. (1989) Survival of recombinant erythropoietin in the circulation: The role of carbohydrates. Blood 73: 84–89. 2910371

[4] Spivak JL, Hogans BB. (1989) The *in vivo* metabolism of recombinant human erythropoietin in the rat. Blood 73: 90–99. 2462945

[5] Tsuda E, Kawanishi G, Ueda M, Masuda S, Sasaki R. (1990) The role of carbohydrate in recombinant human erythropoietin. Eur J Biochem 188: 405–411. 2156701 10.1111/j.1432-1033.1990.tb15417.x

[6] Imai N, Higuchi M, Kawamura A, Tomonoh K, Oh-Eda M, Fujiwara M, et al (1990) Physicochemical and biological characterization of asialoerythropoietin. suppressive effects of sialic acid in the expression of biological activity of human erythropoietin *in vitro* . Eur J Biochem 194: 457–462. 2269277 10.1111/j.1432-1033.1990.tb15639.x

[7] Yamaguchi K, Akai K, Kawanishi G, Ueda M, Masuda S, Sasaki R. (1991) Effects of site-directed removal of *N*-glycosylation sites in human erythropoietin on its production and biological properties. J Biol Chem 266: 20434–20439. 1657925

[8] Wasley LC, Timony G, Murtha P, Stoudemire J, Dorner AJ, Caro J, et al (1991) The importance of *N*- and *O*-linked oligosaccharides for the biosynthesis and *in vitro* and *in vivo* biologic activities of erythropoietin. Blood 77: 2624–2632. 2043765

[9] Delorme E, Lorenzini T, Giffin J, Martin F, Jacobsen F, Boone T, et al (1992) Role of glycosylation on the secretion and biological activity of erythropoietin. Biochemistry 31: 9871–9876. 1390770 10.1021/bi00156a003

[10] Wang YJ, Hao SJ, Liu YD, Hu T, Zhang GF, Zhang X, et al (2010) PEGylation markedly enhances the *in vivo* potency of recombinant human non-glycosylated erythropoietin: A comparison with glycosylated erythropoietin. J Control Release 145: 306–313. 10.1016/j.jconrel.2010.04.021 20427020

[11] Jacobs K, Shoemaker C, Rudersdorf R, Neill SD, Kaufman RJ, Mufson A, et al (1985) Isolation and characterization of genomic and cDNA clones of human erythropoietin. Nature 313: 806–810. 3838366 10.1038/313806a0

[12] Lin FK, Suggs S, Lin CH, Browne JK, Smalling R, Egrie JC, et al (1985) Cloning and expression of the human erythropoietin gene. Proc Natl Acad Sci U S A 82: 7580–7584. 3865178 10.1073/pnas.82.22.7580 PMC391376

[13] Wen D, Boissel JP, Tracy TE, Gruninger RH, Mulcahy LS, Czelusniak J, et al (1993) Erythropoietin structure-function relationships: High degree of sequence homology among mammals. Blood 82: 1507–1516. 8364201

[14] Suliman HB, Majiwa PA, Feldman BF, Mertens B, Logan-Henfrey L. (1996) Cloning of a cDNA encoding bovine erythropoietin and analysis of its transcription in selected tissues. Gene 171: 275–280. 8666286 10.1016/0378-1119(95)00895-0

[15] Fu P, Evans B, Lim GB, Moritz K, Wintour EM. (1993) The sheep erythropoietin gene: Molecular cloning and effect of hemorrhage on plasma erythropoietin and renal/liver messenger RNA in adult sheep. Mol Cell Endocrinol 93: 107–116. 8349021 10.1016/0303-7207(93)90113-x

[16] David RB, Blom AK, Sjaastad OV, Harbitz I. (2001) The porcine erythropoietin gene: cDNA sequence, genomic sequence and expression analyses in piglets. Domest Anim Endocrinol 20: 137–147. 11311851 10.1016/s0739-7240(01)00089-3

[17] Sato F, Yamashita S, Kugo T, Hasegawa T, Mitsui I, Kijima-Suda I. (2004) Nucleotide sequence of equine erythropoietin and characterization of region-specific antibodies. Am J Vet Res 65: 15–19. 14719696 10.2460/ajvr.2004.65.15

[18] McDonald JD, Lin FK, Goldwasser E. (1986) Cloning, sequencing, and evolutionary analysis of the mouse erythropoietin gene. Mol Cell Biol 6: 842–848. 3022133 10.1128/mcb.6.3.842-848.1986 PMC367584

[19] Wang ZL, Chen Y., Yang J, Chen WJ, Zhang YM, Zhao XQ. (2012) cDNA cloning and expression of erythropoietin in the plateau zokor (*Myospalax baileyi*) from the qinghai-tibet plateau. Chin Sci Bull 57: 997–1006.

[20] Vilalta A, Wu D, Margalith M, Hobart P. (2001) Rabbit EPO gene and cDNA: Expression of rabbit EPO after intramuscular injection of pDNA. Biochem Biophys Res Commun 284: 823–827. 11396976 10.1006/bbrc.2001.5028

[21] Nogawa-Kosaka N, Hirose T, Kosaka N, Aizawa Y, Nagasawa K, Uehara N, et al (2010) Structural and biological properties of erythropoietin in *Xenopus laevis* . Exp Hematol 38: 363–372. 10.1016/j.exphem.2010.02.009 20193733

[22] Chou CF, Tohari S, Brenner S, Venkatesh B. (2004) Erythropoietin gene from a teleost fish, *Fugu rubripes* . Blood 104: 1498–1503. 15142879 10.1182/blood-2003-10-3404

[23] Chu CY, Cheng CH, Chen GD, Chen YC, Hung CC, Huang KY, et al (2007) The zebrafish erythropoietin: Functional identification and biochemical characterization. FEBS Lett 581: 4265–4271. 17706649 10.1016/j.febslet.2007.07.073

[24] Paffett-Lugassy N, Hsia N, Fraenkel PG, Paw B, Leshinsky I, Barut B, et al (2007) Functional conservation of erythropoietin signaling in zebrafish. Blood 110: 2718–2726. 17579187 10.1182/blood-2006-04-016535 PMC1988930

[25] Katakura F, Katzenback BA, Belosevic M. (2013) Molecular and functional characterization of erythropoietin of the goldfish (*Carassius auratus L*.). Dev Comp Immunol 40:148–157. 10.1016/j.dci.2013.02.007 23474427

[26] Grodberg J, Davis KL, Sykowski AJ. (1993) Alanine scanning mutagenesis of human erythropoietin identifies four amino acids which are critical for biological activity. Eur J Biochem 218: 597–601. 8269950 10.1111/j.1432-1033.1993.tb18413.x

[27] Wen D, Boissel JP, Showers M, Ruch BC, Bunn HF. (1994) Erythropoietin structure-function relationships. identification of functionally important domains. J Biol Chem 269: 22839–22846. 8077236

[28] Matthews DJ, Topping RS, Cass RT, Giebel LB. (1996) A sequential dimerization mechanism for erythropoietin receptor activation. Proc Natl Acad Sci U S A 93: 9471–9476. 8790354 10.1073/pnas.93.18.9471 PMC38452

[29] Elliott S, Lorenzini T, Chang D, Barzilay J, Delorme E. (1997) Mapping of the active site of recombinant human erythropoietin. Blood 89: 493–502. 9002951

[30] Syed RS, Reid SW, Li C, Cheetham JC, Aoki KH, Liu B, et al (1998) Efficiency of signalling through cytokine receptors depends critically on receptor orientation. Nature 395: 511–516. 9774108 10.1038/26773

[31] Nogawa-Kosaka N, Sugai T, Nagasawa K, Tanizaki Y, Meguro M, Aizawa Y, et al (2011) Identification of erythroid progenitors induced by erythropoietic activity in *Xenopus laevis* . J Exp Biol 214: 921–927. 10.1242/jeb.050286 21346119

[32] Elliott S, Lorenzini T, Asher S, Aoki K, Brankow D, Buck L, et al (2003) Enhancement of therapeutic protein *in vivo* activities through glycoengineering. Nat Biotechnol 21: 414–421. 12612588 10.1038/nbt799

[33] Barrio Rendo ME (1997) Erythropoietin requirement for the maintenance of normal levels of erythropoiesis in the normal adult mouse. Acta Physiol Pharmacol Ther Latinoam 47: 225–228. 9504182

[34] Cowgill LD, James KM, Levy JK, Browne JK, Miller A, Lobingier RT, et al (1998) Use of recombinant human erythropoietin for management of anemia in dogs and cats with renal failure. J Am Vet Med Assoc 212: 521–528. 9491159

[35] MacLeod JN, Tetreault JW, Lorschy KA, Gu DN. (1998) Expression and bioactivity of recombinant canine erythropoietin. Am J Vet Res 59: 1144–1148. 9736393

[36] Randolph JE, Scarlett JM, Stokol T, Saunders KM, MacLeod JN. (2004) Expression, bioactivity, and clinical assessment of recombinant feline erythropoietin. Am J Vet Res 65: 1355–1366. 15524322 10.2460/ajvr.2004.65.1355

[37] Elliott S, Egrie J, Browne J, Lorenzini T, Busse L, Rogers N, et al (2004) Control of rHuEPO biological activity: The role of carbohydrate. Exp Hematol 32: 1146–1155. 15588939 10.1016/j.exphem.2004.08.004

[38] Seyfang A & Jin JH. (2004) Multiple site-directed mutagenesis of more than 10 sites simultaneously and in a single round. Anal Biochem 324: 285–291. 14690693 10.1016/j.ab.2003.10.012

[39] Komatsu N, Yamamoto M, Fujita H, Miwa A, Hatake K, Endo T, et al (1993) Establishment and characterization of an erythropoietin-dependent subline, UT-7/epo, derived from human leukemia cell line, UT-7. Blood 82: 456–464. 8329702

[40] Sebaugh JL. (2011) Guidelines for accurate EC50/IC50 estimation. Pharm Stat 10: 128–134. 10.1002/pst.426 22328315

[41] Gimenez E, de Bolos C, Belalcazar V, Andreu D, Borras E, De la Torre BG, et al (2007) Anti-EPO and anti-NESP antibodies raised against synthetic peptides that reproduce the minimal amino acid sequence differences between EPO and NESP. Anal Bioanal Chem 388: 1531–1538. 17534608 10.1007/s00216-007-1334-8

[42] Darling RJ, Kuchibhotla U, Glaesner W, Micanovic R, Witcher DR, Beals JM. (2002) Glycosylation of erythropoietin affects receptor binding kinetics: Role of electrostatic interactions. Biochemistry 41: 14524–14531. 12463751 10.1021/bi0265022

[43] Pisal DS, Kosloski MP, Balu-Iyer SV. (2010) Delivery of therapeutic proteins. J Pharm Sci 99: 2557–2575. 10.1002/jps.22054 20049941 PMC2857543

[44] Moremen KW, Tiemeyer M, Nairn AV. (2012) Vertebrate protein glycosylation: Diversity, synthesis and function. Nat Rev Mol Cell Biol 13: 448–462. 10.1038/nrm3383 22722607 PMC3934011

[45] Elliott S, Chang D, Delorme E, Eris T, Lorenzini T. (2004) Structural requirements for additional *N*-linked carbohydrate on recombinant human erythropoietin. J Biol Chem 279: 16854–16862. 14757769 10.1074/jbc.M311095200

[46] Wilson S, Felt S, Torreilles S, Howard A, Behan C, Moorhead R, et al (2011) Serum clinical biochemical and hematologic reference ranges of laboratory-reared and wild-caught *Xenopus laevis* . J Am Assoc Lab Anim Sci 50: 635–640. 22330708 PMC3189665

[47] Lasne F & de Ceaurriz J. (2000) Recombinant erythropoietin in urine. Nature 405: 635 10864311 10.1038/35015164

[48] Egrie JC, Dwyer E, Browne JK, Hitz A, Lykos MA. (2003) Darbepoetin alfa has a longer circulating half-life and greater *in vivo* potency than recombinant human erythropoietin. Exp Hematol 31: 290–299. 12691916 10.1016/s0301-472x(03)00006-7

[49] Aizawa Y, Nogawa N, Kosaka N, Maeda Y, Watanabe T, Miyazaki H, et al (2005) Expression of erythropoietin receptor-like molecule in *Xenopus laevis* and erythrocytopenia upon administration of its recombinant soluble form. J Biochem 138: 167–175. 16091591 10.1093/jb/mvi113

